# Effects of Intensity and Fatigue on the Kinetics and Kinematics of the Barbell Squat, Bench Press, and Deadlift in Experienced Lifters: A Systematic Review

**DOI:** 10.1186/s40798-025-00921-x

**Published:** 2025-10-14

**Authors:** Javad A. Bakhshinejad, Jared D. Ramer, Kristen A. Dunsmore, Luke M. Pelton, Lars Berglund

**Affiliations:** 1 Department of Exercise Science, Springfield College, Springfield, MA United States of America; 2 Department of Exercise Science and Health Promotion, Florida Atlantic University, Boca Raton, FL United States of America; 3 Department of Community Medicine and Rehabilitation Umeå, Umeå University, Umeå, 901 87 Sweden

**Keywords:** Powerlifting, Strength, Velocity, Force, Biomechanics

## Abstract

**Background:**

Powerlifting is a competitive strength sport focused on achieving the highest possible single-repetition load in three barbell lifts: the back squat, bench press, and deadlift, each testing maximal force output under standardized conditions. Increases in training intensity and the accumulation of fatigue can lead to measurable alterations in kinetic and kinematic variables, with potential implications for both performance and injury risk. Although trained lifters typically exhibit more stable movement patterns than novices, the biomechanical responses to intensity and fatigue remain complex and variable. This systematic review aimed to identify consistent, observable changes in kinetic and kinematic variables in experienced lifters during the back squat, bench press, and deadlift under conditions of increasing intensity and fatigue.

**Methods:**

A keyword search was performed on MEDLINE Complete, SPORTDiscus, and CINAHL Plus up to June 2024. Studies that examined the effects of load and fatigue on the kinetics and kinematics of the back squat, bench press, and deadlift in experienced lifters were included. The quality of studies was rated according to the Quality Assessment of Observational Cohort and Cross-sectional Studies scale. Results were summarized in tables and with a narrative synthesis.

**Results:**

Twenty-two studies, with a total of 293 participants, met the inclusion criteria. Increased intensity was consistently associated with decreases in mean and peak barbell velocity and power, increased force production, longer concentric durations, and greater joint variability, especially in the acceleration and sticking regions. Fatigue led to similar reductions in velocity and power, although findings on force production were inconsistent across studies. Only two studies examined the deadlift, and reporting practices varied between studies.

**Conclusion:**

Increased intensity and fatigue produce predictable kinetic and kinematic changes in the back squat, bench press, and deadlift, particularly during the acceleration and sticking phases. Velocity consistently decreased with intensity and fatigue, while power and joint mechanics showed greater variability across individuals and studies. Coaches and clinicians should monitor these changes to inform programming and technical interventions. However, different lifters may adopt distinct mechanical strategies as intensity increases, especially near the sticking point. Future research should distinguish between- and within-individual variability in kinetic and kinematic expression and address underrepresented movements, particularly the deadlift.

**Registration:**

This systematic review was registered with PROSPERO (CRD42024550339).

**Supplementary Information:**

The online version contains supplementary material available at 10.1186/s40798-025-00921-x.

## Key Points

• Increases in intensity and fatigue significantly and predictably affect both kinetics and kinematics in the back squat, bench press, and deadlift with an emphasis in the acceleration phase and sticking regions.

• Mean and peak velocity consistently decreased with increasing intensity, while power showed variable responses; joint angles and coordination patterns became more variable at higher intensities, particularly in the sticking region.

• Study quality was generally good, but outcome reporting was inconsistent, and only two studies specifically examined the deadlift under conditions of intensity or fatigue. Additionally, many studies lacked detailed participant recruitment information or control of confounding variables, limiting the generalizability and interpretability of the findings.

**Supplementary Information:**

The online version contains supplementary material available at 10.1186/s40798-025-00921-x.

## Introduction

Powerlifting is a strength sport focused on lifting the heaviest possible load for a single repetition in three barbell movements: the back squat, bench press, and deadlift. Progressive increases in training intensity and fatigue can significantly alter movement mechanics, leading to phase-specific kinematic and kinetic alterations [1]. Each lift consists of four distinct phases: (1) the acceleration region (AR), the initiation of barbell ascent, where bar velocity increases from zero to peak positive velocity; (2) the sticking or failure region (SR), when the bar slows and reaches a local velocity minimum; (3) the maximum strength region (MSR), where bar velocity increases again after the sticking point; and (4) the deceleration region (DR), which concludes with full extension of the hips and knees (back squat, deadlift) or elbows (bench press) [2–5].

Movement execution during each phase of the squat, bench press, and deadlift is influenced by training variables, particularly intensity and fatigue [2, 4, 6]. *Intensity* is typically defined as the percentage of one-repetition maximum (%1RM) [7, 8], though more recent interpretations consider relative intensity, such as proximity to failure, often measured through subjective scales such as rating of perceived exertion (RPE) or repetitions in reserve (RIR) [9–11]. Throughout this review, intensity is used as an umbrella term encompassing both absolute intensity (%1RM) and relative intensity (RPE or RIR). Where relevant, we specify the form of intensity used in each study to maintain clarity.

*Fatigue*, in contrast, refers to a temporary reduction in performance capacity resulting from prior muscular effort [10, 12]. In this review, we focus specifically on neuromuscular fatigue which we operationalize as proximity to failure as a proxy for task-specific fatigue. While the included studies often refer to “fatigue,” most do not measure it directly. Rather, they investigate changes in biomechanics across multiple-repetition sets performed near failure. Therefore, our interpretation is based on the effects of approaching failure, where fatigue is inferred from performance changes (e.g., reductions in barbell velocity, force output, or power) across time points [10, 12]. Accordingly, studies comparing higher repetition sets (e.g., 8-10RM) to lower repetition sets (e.g., 1–3RM), or involving tasks performed to or near failure, were considered to induce greater neuromuscular fatigue [10, 13].

In powerlifting, both increased intensity and fatigue have been shown to impact kinematics (e.g., joint angles, velocity, bar path) [14, 15] and kinetics (e.g., force output, joint moments) [16–19]. With increased intensity or fatigue, concentric velocity decreases and joint mechanics change—for example, excessive forward lean and spinal flexion in the squat or deadlift, or an exaggerated bar path toward the shoulder in the bench press [3, 20–23]. These adjustments can, in turn, alter barbell path, contributing to horizontal or vertical displacement [4, 12]. However, inconsistencies exist both between individuals and between trained and untrained participants, with trained individuals generally maintaining more consistent technique under higher intensities and fatigue [24, 25]. Therefore, findings from studies on untrained participants may not generalize to trained lifters [15, 26], highlighting the need to systematically review and synthesize this literature in trained populations.

Accordingly, the primary objective of this systematic review was to synthesize current research on the effects of intensity and fatigue on the kinematic and kinetic characteristics of performance of the barbell back squat, bench press and deadlift in experienced lifters by addressing the question: “How do intensity and fatigue affect the kinetics and kinematics of the barbell back squat, bench press, and deadlift in experienced lifters?”

## Methods

The methodology and reporting were created in accordance with Preferred Reporting Items for Systematic Reviews and Meta-Analyses (PRISMA) [27]. The review was pre-registered on the International Prospective Register of Systematic Reviews (PROSPERO) prior to the search process (registration number: CRD42024550339). The pre-registered search protocol did not capture all relevant studies. Therefore, we supplemented our approach with backward and forward citation searches of the included studies, along with hand searches in Google Scholar, to ensure the comprehensiveness of our search.

### Eligibility Criteria

Original peer-reviewed empirical studies were included in this review. There were no limitations on publishing year. The following criteria had to be met for inclusion: cross-sectional research studies that assessed powerlifting exercises (back squat, bench press, or deadlift), while including either a kinetic or kinematic assessment at different absolute intensities (%1RM), relative intensities (e.g., RIR or RPE), proximity-to-failure, or fatigue. The sample population had to consist of apparently healthy, non-injured individuals over 18 years old with at least one year of resistance training experience, classified as a competitive powerlifter, or a strength level corresponding to advanced resistance training status as outlined by Santos Junior et al. [28].

Any studies that had aims unrelated to immediately apparent kinematic or kinetic changes of the barbell back squat, bench press, or deadlift in response to varying intensities or levels of fatigue were excluded; these included reliability/validity studies due to their focus on measurement properties rather than fatigue or intensity; longitudinal training studies that observe changes over extended periods of time, rather than immediate mechanical changes; non-free-weight variations and partial-depth squats which differ mechanically from standard movements; studies manipulating rest lengths, repetition tempo, or set configuration (i.e. cluster sets, accentuated eccentric loading, super-sets, drop sets, etc.) which affect movement patterns differently than focusing on intensity or fatigue; population comparison studies that involve multiple groups rather than trained individuals alone; and technical variation studies that compare different movement strategies rather than strictly examining intensity or fatigue effects.

### Search Strategy

The literature search was performed from 06/15/2024 to 06/30/2024 by searching the following databases: MEDLINE Complete, SPORTDiscus, and CINAHL Plus. These databases were searched using the following search terms and phrases and Boolean operators: (1) resistance train* OR strength train* OR weight train* OR resistance exercise OR powerlift* OR weightlift* (2) squat* OR bench* OR deadlift* (3) kinematics OR kinetics OR biomechanics (4) load OR intensity OR RPE OR rating of perceived exertion OR perceived exertion OR borg scale. Two authors (JB and LB) performed a double-blind screening of titles, abstracts, and full texts. Upon completion of the screening process JB and LB compared results and determined the final studies that were selected for inclusion. An additional screening was performed by JB to ensure that any relevant studies were not excluded. Backward and forward reference screening was conducted, and an additional hand search using the phrase “kinetics and kinematics at different intensities” was performed in Google Scholar to ensure all relevant articles were identified.

### Data Extraction

Each of the studies were read and coded by one author (JB) for the following variables: exercise (e.g., back squat, bench press, deadlift), participant characteristics (e.g., sample size, height, body mass, training status), intervention details (e.g., fatigue or intensity assessment), session characteristics (e.g., sets, reps, load, rest), outcomes, and statistical results. For each study, the statistical analysis performed was extracted and their respective statistical findings were reported.

### Quality Assessment

Two authors (JB and LB) performed a double-blind evaluation of the methodological study quality using the Quality Assessment Tool for Observational Cohort and Cross-Sectional Studies [29]. The evaluation of each study accounted for the following factors: whether the research question or objective was clearly articulated; if the study population was well-defined and specified; if the participation rate of eligible individuals was at least 50%; if all subjects were selected or recruited from comparable populations, and if inclusion and exclusion criteria were uniformly applied to all participants. Additionally, it was considered whether a sample size justification, power description, or variance and effect estimates were provided; if the exposures of interest were measured before the outcomes; if the timeframe was adequate to expect an association between exposure and outcome; if varying levels of exposure were examined; if exposure measures were clearly defined, valid, reliable, and consistently implemented; if the exposure was assessed more than once over time; if outcome measures were clearly defined, valid, reliable, and consistently implemented; if outcome assessors were blinded to the exposure status of participants; if loss to follow-up after baseline was 20% or less; and if key potential confounding variables were measured and statistically adjusted for.

Each of the studies were evaluated as good: meeting ≥ 6 criteria, fair: meeting 4–5 criteria, and poor: meeting ≤ 3 criteria. Study findings were interpreted in relation to their quality ratings, where results from ‘fair’ or ‘poor’ quality studies were contextualized with consideration of their methodological limitations.

### Data Synthesis

An overall narrative synthesis was presented in relation to the study question. No additional assumptions or calculations regarding the data reported in the included studies were made. Data were presented in tables summarizing study characteristics and the reported outcomes for each individual exercise (back squat, bench press, and deadlift). Figures were created to visually display a summary of the results of the review. A descriptive summary and narrative synthesis were performed as motivated by the presence of heterogeneity in the outcomes for the included studies. Findings were interpreted in the context of each study’s methodological quality, with greater weight given to results from studies rated as “good” and more cautious interpretation applied to findings from those rated “fair” or “poor.”

## Results

**Fig. 1:**
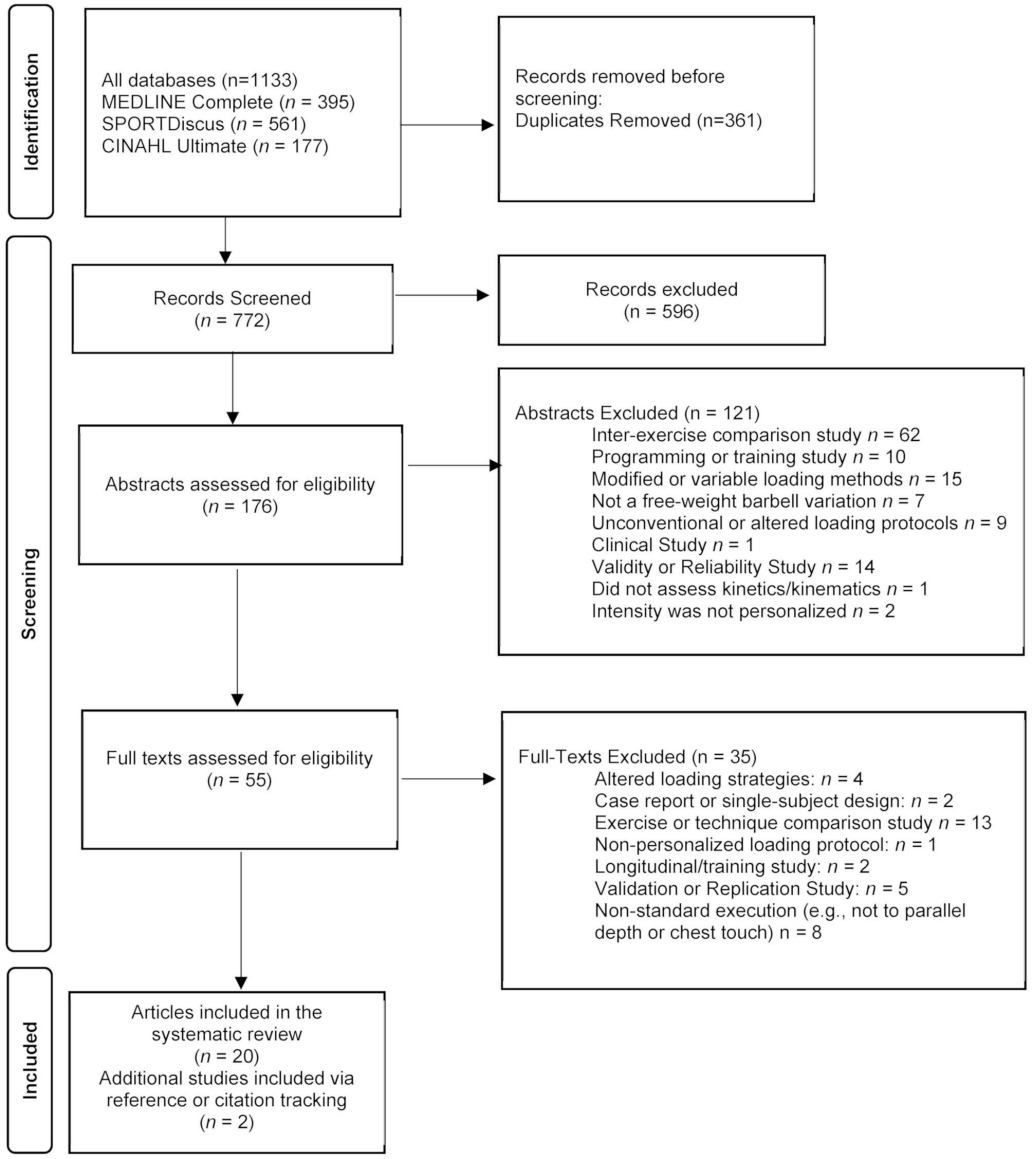
Flow diagram of the literature screening according to the Preferred Reporting Items for Systematic Reviews and Meta-Analyses (PRISMA)

The systematic literature search yielded a total of 1133 potentially relevant research studies which were screened for their eligibility and the results are presented in Fig. 1. After the full screening process, 20 studies met the inclusion criteria for this review, while two additional studies were identified via hand searching from: (1) Google Scholar (phrase: “kinetics and kinematics at different intensities”) [30], and (2) references of included studies [20].

### Study Characteristics

The study characteristics are presented in Table 1. Twenty-two cross-sectional cohort studies were included in the review, each of which examined the free-weight straight barbell variation of the back squat [4, 14, 16, 17, 20, 26, 31–36], bench press [3, 18, 23, 30, 37–40], or deadlift [41, 42]. Studies by Wilson et al. [3] and Elliot et al. [23] were based on the same data. A total of 293 resistance-trained participants (males: *n* = 256; females: *n* = 37) were included in this review. Three included studies [16, 33, 34] used strength-based criteria to define experience (squatting 1.2 or 1.5 times bodyweight) rather than years of training, while two studies [3, 23] recruited specifically competitive powerlifters. Only two studies [41, 42] specifically examined the DL, and both focused solely on intensity without incorporating fatigue protocols, limiting the scope of DL-specific conclusions.

**Table 1:** Characteristics of the included studies examining the barbell Squat, bench Press, and deadlift

Reference	Aim(s)	Exercise	Total Sample Size (*N*)	Men (*n*)	Women (*n*)	Age (years)	Height (cm)	Weight (kg)	Resistance-training experience (years)
Brice et al. [16]	The purpose of this study was to examine how performing multiple sets to volitional failure alters ankle, knee, hip, and lumbo-pelvis kinetics and kinematics under a moderate-heavy load (i.e., 80% 1RM).” The study aimed to examine the impact of performing multiple sets to failure on squatting mechanics.	SQ	11	11	-	26.2 ± 3.8	178 ± 8	82.4 ± 8.9	>1.5x bw SQ
Bryanton et al. [17]	The primary aim of this study is to investigate the effects of barbell load and squat depth on the relative muscular effort (RME) of the hip extensors, knee extensors, and ankle plantar flexors during the squat exercise.	SQ	10	-	10	22.5 ± 2.1	167.1 ± 5.1	62.5 ± 6.5 kg	>1
Carroll et al. [26]	(1) To examine the variation in velocity and power with increasing intensity in the back squat among subjects; and (2) To explore individual subject characteristics as possible explanations for variations of velocity in the back squat.	SQ	14	14	-	25.0 ± 2.6	178.9 ± 8	88.2 ± 15.8	>1
Falch et al. [33]	The primary aim of this study is to compare kinematics and electromyography (EMG) in the lower extremities during the concentric phase of the last repetition when performing the bilateral back squat at different repetition maximums (1, 3, 6, and 10RM).	SQ	13	13	-	23.6 ± 1.9	175.3 ± 5.5	82.2	>1.2x bw
Kipp et al. [32]	To determine muscle-specific contributions to lower extremity NJMs during squats with different external loads.	SQ	9	9	-	21.8 ± 0.1	182 ± 0.06	81.5 ± 6.3	>1
Kubo et al. [36]	Whether a deceleration sub-phase occurs during SQ with different loads and to assess the influence of load on the deceleration sub-phase duration and negative impulse during the deceleration subphase.	SQ	16	16	-	25 ± 3	173 ± 7	83.2 ± 16.1	>1
Larsen et al. [34]	The purpose of this study was to investigate the effects of 90%, 100%, and 102% of 1-RM barbell loads on kinematics, kinetics, and myoelectric activity in back squats.	SQ	12	12	-	27.3 ± 3.8	180.3 ± 6.7	83.5 ± 7.8	>1.5x bw
Maddox et al. [4]	To examine coordination of the thigh and shank, trunk and thigh, and the hip and knee during the concentric phase of maximum, supra-maximum (at 105% max), and sub-maximum (at 80% max) back squats.	SQ	14	7	7	25.2 ± 4.1	169 ± 11	80.67 ± 12.64	>1
Maddox & Bennett [20]	The purpose of this study was to determine how loading affects performance (whole-body velocity and acceleration) and joint level biomechanics (angles and moments) during the acceleration and sticking regions of back squats.	SQ	20	10	10	18–55	-	80.53 ± 14.28	>1
Van Den Tillaar, et al. [31]	To compare barbell kinematics and muscle patterning in free-weight back squatting with different loads, but with maximum lifting velocity, in young males with resistance training experience.	SQ	10	10	-	27.3 ± 5.9	178	82.8 ± 16.6	>1
Vasquez et al. [35]	Assess the effect of resistance exercise performed to volitional failure on RPE using power as an indication of fatigue.	SQ	12	12	-	21.9 ± 1.3	177.9	77.8 ± 8.0	>2
Weakley et al. [14]	Assess the effects of 10%, 20%, and 30% velocity loss thresholds on kinetic, kinematic, and repetition characteristics in the free-weight back squat.	SQ	16	16	-	23.1 ± 2.4	180	88.8 ± 13.3	>2
Franco-García et al. [30]	To identify the mechanism(s) during the bench press responsible for the previously reported sticking region phenomenon.	BP	27	17	10	men = 21.4 ± 1.5; women = 21.7 ± 2.3	men = 175.1 ± 6.7 women = 163.3 ± 10.8	men = 75.8 ± 7.7 women = 57.2 ± 6.8	>1
Król and Golaś [40]	(1) To investigate the internal and external structures of the flat bench press. (2) To investigate the relationships that characterize these structures, according to the bench press load.	BP	20	20	-	24.7 ± 0.9	177 ± 0.08	80.2 ± 8.6	>1
Larsen et al. [18]	To investigate the effects of 1-RM, 3-RM, 6-RM, and 10-RM on kinematics and electromyographic activity in the barbell bench press during the last repetition.	BP	12	12	-	23.5 ± 2.6	183.8 ± 4.2	84.3 ± 7.8	>1
Mangine et al. [37]	The purpose of this study was to examine the effects of using RIR or RM prescription during an acute bout of bench press on total training volume, performance recovery, perceived effort, and muscle damage in resistance trained men.	BP	10	10	-	24.6 ± 3.0	176 ± 5	85.7 ± 14.0	>1
Van Den Tillaar and Saeterbakken [39]	To examine the effect of fatigue during one set of 6RM bench pressing upon the muscle patterning and performance (kinematics of the 6 repetitions) in experienced resistance-trained subjects.	BP	14	14	-	22.5 ± 2.0	182 ± 0.07	82.0 ± 7.8	>1
Van Den Tillaar and Sousa [38]	To analyze shoulder kinematics symmetry at different load intensities, considering full range of motion, velocities, and accelerations during eccentric and concentric phases of bench press, and compare these variables between different load intervals.	BP	13	13	-	24.2 ± 2.0	178	81.5 ± 9.1	>1
Elliott et al. [23]	To compare barbell kinematics and muscle patterning in bench press with different loads, but with maximum effort, in young males with resistance training experience.	BP	10	10	-	-	-	-	Competitive Powerlifters
Wilson et al. [3]	To analyze the data from the study by Elliott et al. [23] so that the bar path and force-profile characteristics of a single repetition maximum and submaximal (81% of maximal load) bench press, performed by elite powerlifters, could be compared.	BP	10	10	-	-	-	-	Competitive Powerlifters
Blatnik et al. [41]	Determine a more comprehensive power–load curve for the deadlift exercise and to establish the loads that optimize power for the bar, body, and system (bar + body).	DL	8	8	-	22 ± 2.38	180 ± 5	88.97 ± 14.88	>2
Lawson et al. [42]	Identify whether there is an optimum load in relation to peak force development and RFD in the straight bar deadlift and to examine whether baseline strength levels influence this optimum load.	DL	12	12	-	25.1 ± 5.4	177 ±: 11	81.5 ± 12.5	>2
Total:			293	256	37				

Note. SQ = Barbell Back Squat; BP = Barbell Bench Press; DL = Barbell Deadlift, bw = Bodyweight Strength, RFD = Rate of Force Development, RM = Repetition Maximum, RIR = Repetitions in Reserve, NJM = Net Joint Moment

Each of the studies were assessed using the Quality Assessment Tool for Observational Cohort and Cross-Sectional Studies (see Table 2) [29]. Seventeen studies were rated as “good” quality (meeting ≥ 6 criteria), while five were rated as “fair” [4, 20, 36] or “poor” [3, 23] due to limitations such as unclear recruitment strategies, lack of sample size justification, or insufficient reporting of outcome measures. Only one study [14] controlled for confounding variables, and just six studies [16, 17, 30, 33, 37, 42] reported a priori sample size calculations or power calculations. Seven studies [14, 30, 31, 34, 35, 38, 42] clearly reported comprehensive participant selection criteria and recruitment strategies. None of the included studies employed blinding of outcome assessors or assessed exposures more than once over time, which may have introduced performance and detection biases. These limitations highlight potential threats to internal and external validity across studies and suggest the need for caution when generalizing findings, especially for underrepresented lifts such as the deadlift.

**Table 2:** Risk of bias assessment using the quality assessment of observational cohort and Cross-sectional studies scale

Criteria/Included Studies	Blatnik et al. [41]	Brice et al. [16]	Bryanton et al. [17]	Carroll et al. [26]	Elliott et al. [23]	Falch et al. [33]	Franco-García et al. [30]	Kipp et al. [32]	Król and Golaś [40]	Kubo et al. [36]	Larsen et al. [34]	Larsen et al. [18]	Lawson et al. [42]	Maddox et al. [4]	Maddox & Bennett [20]	Mangine et al. [37]	Van Den Tillaar et al. [31]	Van Den Tillaar and Saeterbakken [39]	Van Den Tillaar and Sousa [38]	Vasquez et al. [35]	Weakley et al. [14]	Wilson et al. [3]
1. Was the research question or objective in this paper clearly stated?	Y	Y	Y	Y	Y	Y	Y	Y	Y	Y	Y	Y	Y	Y	Y	Y	Y	Y	Y	Y	Y	Y
2. Was the study population clearly specified and defined?	Y	Y	Y	Y	Y	Y	Y	Y	Y	Y	Y	Y	Y	Y	Y	Y	Y	Y	Y	Y	Y	Y
3. Was the participation rate of eligible persons at least 50%?	CD	CD	CD	CD	CD	CD	CD	CD	CD	CD	CD	CD	CD	CD	CD	CD	CD	CD	CD	CD	CD	CD
4. Were all the subjects selected or recruited from the same or similar populations (including the same time period)? Were inclusion and exclusion criteria for being in the study prespecified and applied uniformly to all participants?	CD	Y	CD	CD	CD	Y	Y	Y	Y	CD	CD	Y	Y	CD	CD	Y	Y	Y	Y	Y	Y	CD
5. Was a sample size justification, power description, or variance and effect estimates provided?	N	Y	Y	N	N	Y	Y	N	N	N	Y	N	Y	N	N	Y	N	N	N	N	N	N
6. For the analyses in this paper, were the exposure(s) of interest measured prior to the outcome(s) being measured?	NA	NA	NA	NA	NA	NA	NA	NA	NA	NA	NA	NA	NA	NA	NA	NA	NA	NA	NA	NA	NA	NA
7. Was the timeframe sufficient so that one could reasonably expect to see an association between exposure and outcome if it existed?	NA	NA	NA	NA	NA	NA	NA	NA	NA	NA	NA	NA	NA	NA	NA	NA	NA	NA	NA	NA	NA	NA
8. For exposures that can vary in amount or level, did the study examine different levels of the exposure as related to the outcome (e.g., categories of exposure, or exposure measured as continuous variable)?	Y	Y	Y	Y	Y	Y	Y	Y	Y	Y	Y	Y	Y	Y	Y	Y	Y	Y	Y	Y	Y	Y
9. Were the exposure measures (independent variables) clearly defined, valid, reliable, and implemented consistently across all study participants?	Y	Y	Y	Y	Y	Y	Y	Y	Y	Y	Y	Y	Y	Y	Y	Y	Y	Y	Y	Y	Y	Y
10. Was the exposure(s) assessed more than once over time?	NA	NA	NA	NA	NA	NA	NA	NA	NA	NA	NA	NA	NA	NA	NA	NA	NA	NA	NA	NA	NA	NA
11. Were the outcome measures (dependent variables) clearly defined, valid, reliable, and implemented consistently across all study participants?	Y	Y	Y	Y	N	Y	Y	Y	Y	Y	Y	Y	Y	Y	Y	Y	Y	Y	Y	Y	Y	N
12. Were the outcome assessors blinded to the exposure status of participants?	N	N	N	N	N	N	N	N	N	N	N	N	N	N	N	N	N	N	N	N	N	N
13. Was loss to follow-up after baseline 20% or less?	NA	NA	NA	NA	NA	NA	NA	NA	NA	NA	NA	NA	NA	NA	NA	NA	NA	NA	NA	NA	NA	NA
14. Were key potential confounding variables measured and adjusted statistically for their impact on the relationship between exposure(s) and outcome(s)?	N	N	N	Y	N	N	N	N	N	N	N	N	Y	N	N	N	N	N	N	N	Y	N
Quality Rating	Good	Good	Good	Good	Poor	Good	Good	Good	Good	Fair	Good	Good	Good	Fair	Fair	Good	Good	Good	Good	Good	Good	Poor

Note. CD = Cannot determine; N = No; Y = Yes; NA = Not Applicable. Good = meets ≥ 6 criteria, fair = meets 4–5 criteria; poor = meets ≤ 3 criteria

### Relationship between Intensity and Kinematics and Kinetics

**Fig. 2:**
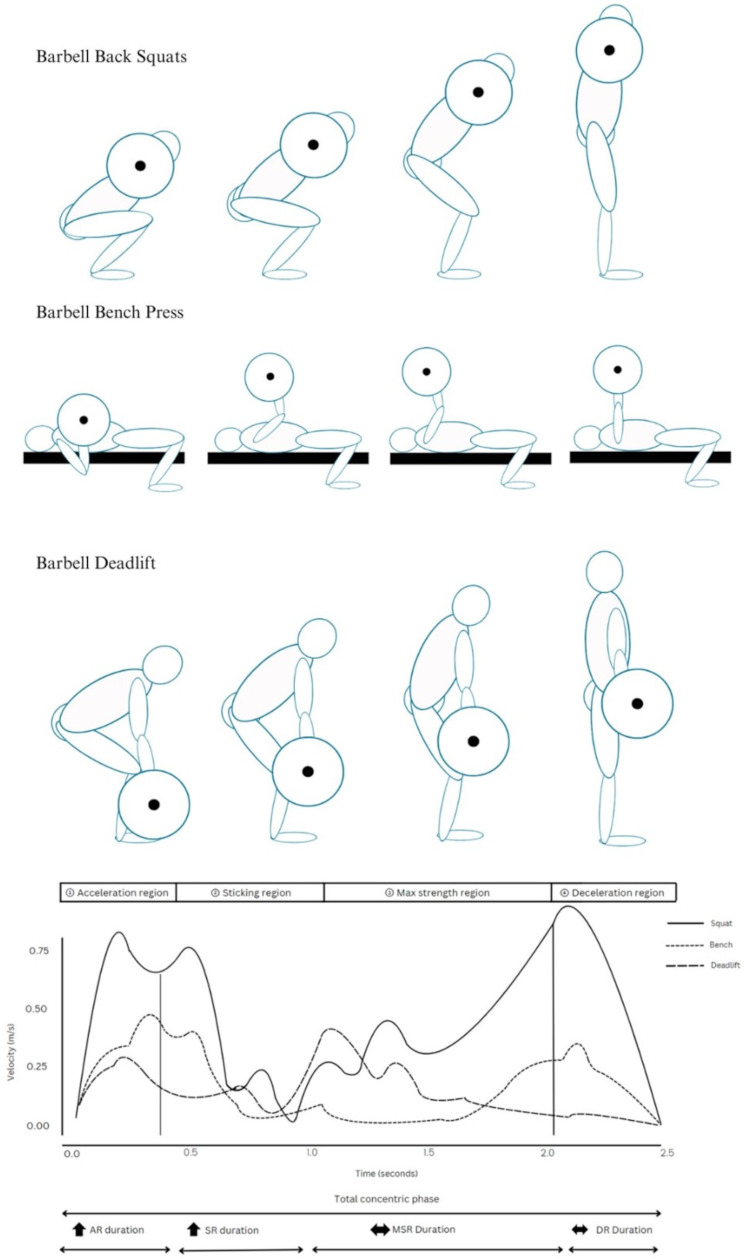
Illustration of the effects of increased intensity on velocity changes at the (1) Acceleration Region (AR), (2) Sticking Region (SR), (3) Maximum Strength Region (MSR), and (4) Deceleration region (DR) of the Barbell Back Squat, Barbell Bench Press, and Barbell Deadlift. Arrows indicate: ↑ = Increased Relative Duration, ↔ = Unchanged Relative Duration. The velocity-time curves were adapted with permission each from publications by Escamilla et al. [2, 5] and Lockie et al. [6]

The findings for each lift (back squat, bench press, and deadlift) are summarized in Fig. 2; Tables 3, 4 and 5.

**Table 3:** Study outcomes for increases in intensity and fatigue in the barbell back squat

Reference	*n*	Intensity Measurement	Fatigue Measurement	Analysis	Intensity (i.e., Reps x %1RM)	Rest Interval (minutes)	Kinetic Outcomes	Kinematic Outcomes
Brice et al. [16]	11	%1RM	Between Repetitions	Paired T-Test	3 x AMRAP 80% (Second vs. Final repetition)	2	Mean joint moments: Knee: ↑↑↑ (*d* = 0.90–1.23) Hip: ↑↑ (*d* = 0.73) Lumbo-pelvis: ↑↑ (d = 0.72) Mean power: Hip: ↓↓↓ (*d* = 1.67) Knee: ↓↓↓ (*d* = 1.50)	↓↓↓ MCV (*d* = 1.05) Angular MCV: Hip: ↓↓↓ (*d* = 1.35) Knee: ↓↓↓ (*d* = 1.26 Ankle: ↓↓↓ (*d* = 1.14) ↔ ROM at ankle, knee, and hip
Bryanton et al. [17]	10	%1RM and RIR	x	One-way RM ANOVA	1 × 50% 1 × 60% 1 × 70% 1 × 80% 1 × 90%	3–5	NJM: Ankle: ↑ Plantar flexor* Hip: ↑ Extensor* Knee: ↔ Extensor*	↓ MCV*
Carroll et al. [26]	14	%1RM	x	Pearson product-moment zero-order correlation	2 × 30% 2 × 40% 2 × 50% 2 × 60% 2 × 70% 2 × 80% 2 × 90%	3	↓↓↓ Mean Power (r^2^ = 0.80) ↑ Between-Subject Variation of Mean Power (CV = 19–64)*	↓↓↓ MCV (r^2^ = 0.80) ↑ Between-Subject Variation of MCV (CV = 10–55)*
Falch et al. [33]	13	1RM, 3RM, 6RM, and 10RM	Last Repetition Between each RM	One-way ANOVA + RM ANOVA	1RM 3RM 6RM 10RM (Last Rep)	>5	x	↓↓↓ Trunk lean at AR(η_p_^2^ ≥ 0.25) ↓↓↓ Knee flexion at SR (η_p_^2^ ≥ 0.25) ↔ Hip angle (η_p_^2^ ≤ 0.15) ↔ MCV (η_p_^2^ ≤ 0.15)
Kipp et al. [32]	9	%BW	x	SPM ANOVA	0% x 4 25% x 4 50% x 4 75% x 4 100% x 4	x	NJM: ↑ Hip Extension* ↑ Knee Extension* ↑ Ankle Plantarflexion* Muscle Moments ↑GM, Adductor Magnus, Hamstrings*	Joint Ratios: ↔ Hamstring: GM at hip* ↔ Vastii: Hamstring at knee*
Kubo et al. [36]	16	%1RM	x	RM ANOVA	1 × 0% 1 × 12% 1 × 27% 1 × 42% 1 × 56% 1 × 71% 1 × 85% (Random order)	5	x	↓↓↓ Relative Duration of Deceleration Subphase (η^2^ = 0.86) ↑↑↑ Duration of Concentric Phase (η^2^ = 0.82) ↑ Absolute Duration of Deceleration Subphase (η^2^ = 0.05) ↑↑↑ Acceleration Subphase Duration (η^2^ = 0.85)
Larsen et al. [34]	12	%1RM	x	RM ANOVA	90% 100% 102%	4	NJM: ↑↑↑ Hip in the AR ↓↓↓ Knee in the AR + SR (η^2^ ≥ 0.60) ↑↑↑ Hip Extension (η^2^ ≥ 0.68) ↔ Knee Extension (η2>0.075) ↔ Ankle (η^2^ ≤ 0.24) ↔ Ankle Plantarflexion (η^2^ = 0.021)	↓↓↓ MCV (η^2^ = 0.87) ↑↑↑ Barbell displacement (η^2^ = 0.82) ↔ Timing in initiation of SR + ↑↑↑ Delayed Second half of SR (η^2^ = 0.28) ↑↑↑ Torso inclination + ankle dorsiflexion angle in SR ↑↑↑ Hip flexion in SR (90% & 100% loads only) ↑↑↑ Hip abduction in AR (all loads) + ↓↓↓ in SR (90% & 100%) ↑↑↑ Knee flexion in SR (90% & 100% loads only) ↑↑↑ Knee abduction in AR (90% & 100% loads only)
Maddox et al. [4]	14	%1RM	x	One-way RM MANOVA (condition x intensity) 24 within-subjects ANOVAs	1 × 80% 1 × 100% 1 × 105%	10	x	↑↑↑ Effect on coupling angles (η_p_^2^ = 0.26–0.50) ↑↑↑ Variability in Upper Body Mechanics (η_p_^2^ = 0.28–0.39) ↑↑↑ Variability in knee-hip moment couplings at SR (η_p_^2^ = 0.25–0.27) All intensities = Strong focus on knee extension at SR (η_p_^2^ = 0.50)
Maddox & Bennett [20]	20	%1RM	x	Statistical parametric mapping RM ANOVA	1 × 80% 1 × 100% 1 × 105%	10	NJM: ↑ Knee Extension* (105% vs. 100% + 80%) ↑ Hip Extension* (105% + 100% vs. 80%) ↑ Ankle eversion* (100% vs. 105% + 80%) ↓ Hip Abduction* (105% vs. 80%) Joint Contribution To Support Moment ↓ Knee + ↑ Hip	↓ MCV ↓ Vertical Acceleration Joint Angle Changes: ↓ Ankle dorsiflexion + eversion ↑ Knee Flexion* (105 vs. 100% and 80%) at SR ↓ Knee Flexion* (100% vs. 80%) at SR ↓ Hip flexion (100% vs. 105% + 80%) ↓ Hip Abduction (100% vs. 80%)
Van Den Tillaar et al. [31]	10	%1RM	x	EMG: RM ANOVA Kinematics: Two-way RM ANOVA (muscles x intensity)	2 × 30% 2 × 40% 2 × 50% 2 × 60% 1 × 70% 1 × 80% 1 × 90% 1 × 100%	3–5	x	↔ Average Lowering Velocity* ↓ Mean Velocity* ↓ Peak Velocity* ↑↑↑ Upward Phase Duration (η^2^ = 0.84) ↑ Later Peak Velocity Duration*
Vasquez et al. [35]	12	%1RM	Varying Intensities to Failure	RM ANOVA	3 × 50%+ 3 × 70%+ 3 × 90%+ (2 sets + 1 set to failure)	10	↑ Intensity + ↑ Velocity Loss = ↓↓↓ Peak Power of last repetition (η^2^ = 0.53) ↑ Intensity + Velocity Loss = ↓↓↓ Repetitions (η2 = 0.91)	x
Weakley et al. [14]	16	MCV	Varying Proximities to Failure	LMM (fixed = velocity loss, random intercept = athlete, random slope = set)	5 × 0.70 m/s, 3 sessions; terminate when velocity drops by 10%, 20%, or 30%	3	↑ Velocity Loss = ↔ Mean or Peak Force* ↑ Velocity Loss = ↓ Power* ↑ Velocity Loss = ↓ Mean Set Power*	↑ Velocity Loss = ↓ Peak Velocity* ↑ Velocity Loss = ↑ Repetitions Performed*
Total	157							

Note. 1RM = 1 Repetition Maximum; MCV = Mean Concentric Velocity; BW = Bodyweight; RPE = Rating of Perceived Exertion; AR = Acceleration Region; NJM = Net Joint Moment; SR = Sticking Region; RM = Repeated Measures; ANOVA = Analysis of Variance; MANOVA = Multivariate Analysis of Variance; Linear Mixed Effects Model = LMM, CV = Coefficient of Variability; Changes in variables are indicated as ↓ = decreased, ↔ = no change, ↑ = increased. Effect size magnitudes were classified as small (η2, ηp2 = 0.01; r2 = 0.02), medium (η2, ηp2 = 0.06; r2 = 0.13), and large (η2, ηp2 = 0.14; r2 = 0.26). Magnitude of effect is demonstrated ↓ or ↑ = Small Effect, ↓↓ or ↑↑ = Moderate Effect, ↓↓↓ or ↑↑↑ = Large Effect; Effect Size = ES

* = study presented no effect size for the finding

**Table 4:** Study outcomes for increases in intensity and fatigue in the barbell bench press

Reference	*n*	Intensity Measurement	Fatigue Measurement	Analysis	Intensity (i.e., Reps x %1RM)	Rest Interval (minutes)	Kinetics Outcomes	Kinematic Outcomes
Elliott et al. [23]	10	%1RM	x	Not reported	1 × 80% 1 × 100% 1 × 105%	5–8	↔ Shoulder + Elbow Moment Arm Changes*	↑ SR Duration* ↑ Horizontal Displacement*
Larsen et al. [18]	12	1-RM, 3-RM, 6-RM, and 10-RM	Varying RMs	EMG: ANOVA (RM x region) Kinematics: RM ANOVA (RM x event)	1-RM 3-RM 6-RM 10-RM	4	x	↓↓↓ Velocity at first peak and max velocity phase (η_p_^2^ ≥ 0.26) ↔ Horizontal and vertical displacement* ↔ Shoulder flexion, abduction, and elbow extension angles* ↔ Peak elbow and shoulder abduction velocity*
Franco-García et al. [30]	27	%1RM	x	RM Two-way ANOVA (laterality x intensity)	5–10 easy x3-5 x2-3 x1 increasing weight	4–5	x	↓↓↓ Max (η_p_^2^ = 0.54) and Mean (η_p_^2^ = 0.32) Acceleration ↓↓↓ Mean (η_p_^2^ = 0.49) and Max (η_p_ = 0.54) Velocity ↔ Laterality at max and mean acceleration (*p* > .05) ↑↑↑ Laterality at max (η_p_^2^ = 0.19) and mean (η_p_^2^ = 0.16) velocity
Król and Golaś [40]	20	%1RM	x	MANOVA	1 × 70% 1 × 80% 1 × 90% 1 × 100%	5		↓ Mean and Max Acceleration ↓ Mean and Max Velocity Ascent and Descent Phase: ↑ Vertical and Horizontal displacement
Mangine et al. [37]	14	%1RM	Varying Proximities to Failure	Two-way ANOVA	5 x AMRAP x 80% 5 × 3RIR x 80%	3–5	↑ reps = ↓↓↓ Work (Force x Displacement; ηp² = 0.77)	↓↓↓ MCV (η_p_^2^ = 0.75)
Van Den Tillaar and Saeterbakken [39]	14	6RM	Initial and Final Repetition Comparison	One-way ANOVA	6-RM (Rep 1–6 Comparison)	4	x	Between 1–6 Repetitions: ↓ Starting Position; ↑↑↑ SR, MSR + ↓↓↓ AR Duration (η2 = 0.33–0.54) ↓↓↓ Minimum Velocity + ↓↓↓ Peak Velocity (η2 ≥ 0.40) ↓↓↓ Distance of barbell at peak velocity ↔ Position of barbell at minimum velocity
Van Den Tillaar and Sousa [38]	13	%1RM	x	RM Two-way ANOVA (phase x intensity)	30–100% (10% increments), 2 reps (30–60%), 1 rep (70–100%)	3–5	x	↓↓↓ Mean and Peak Velocity (η^2^ ≥ 0.32) ↑↑↑ Descending and Ascending Lifting Time (η^2^ ≥ 0.33) ↑↑↑ Descending and Ascending Lifting Distance (η^2^ ≥ 0.36)
Wilson et al. [3]	10	%1RM	x	RM One-way ANOVAs (intensity)	1 × 80% 1 × 100% 1 × 105%	5–8	x	↑ AR, SR Relative Times (Concentric) * ↓ Max Strength Phase and Deceleration Phase Relative Times (Eccentric) * ↑ Horizontal Displacement towards the Shoulder*
Total	120							

Note. 1RM = 1 Repetition Maximum; SR = Sticking Region; RM = Repeated Measure; NJM = Net Joint Moments; ANOVA = Analysis of Variance; Effect size magnitudes were classified as small (η2, ηp2 = 0.01; r2 = 0.02), medium (η2, ηp2 = 0.06; r2 = 0.13), and large (η2, ηp2 = 0.14; r2 = 0.26). Changes in variables are indicated as ↓ = decreased, ↔ = no change, ↑ = increased. Magnitude of effect is demonstrated ↓ or ↑ = Small Effect, ↓↓ or ↑↑ = Moderate Effect, ↓↓↓ or ↑↑↑ = Large Effect

* = study presented no effect size for the finding

**Table 5:** Study outcomes for increases in intensity in the barbell deadlift

Reference	*n*	Intensity Measurement	Fatigue Measurement	Analysis	Intensity (i.e., Reps x %1RM)	Rest Interval (minutes)	Kinetics Outcomes	Kinematic Outcomes
Blatnik et al. [41]	8	%1RM	x	RM MANOVA (dependent variable x intensity)	2 × 30% 2 × 40% 2 × 50% 2 × 60% 2 × 70% 2 × 80% 2 × 90%	5	↑↑↑ System (Body + Bar) Peak Power (η^2^ = 0.53) ↓↓↓ Peak Power Body (η^2^ = 0.52) and Bar (η^2^ = 0.67) ↓↓↓ Peak Body Force (η^2^ = 0.63) ↑↑↑ Peak System (Body + Bar) Force (η^2^ = 0.93)	↓↓↓ Peak Velocity of Bar (η^2^ = 0.92), Body (η^2^ = 0.80), and System (η^2^ = 0.68)
Lawson et al. [42]	12	%1RM	x	RM ANOVA	3 × 20% 3 × 30% 3 × 40% 3 × 50% 3 × 60% 3 × 70% 3 × 80% 3 × 90%	3–5	↔ RFD ↑↑↑ Peak Force (η^2^ = 0.75)	x
Total	20							

Note. 1RM = 1 Repetition Maximum; MANOVA = Multivariate Analysis of Variance; ANOVA = Analysis of Variance; RM = Repeated Measures; RFD = Rate of Force Development. Effect size magnitudes were classified as small (η2, ηp2 = 0.01; r2 = 0.02), medium (η2, ηp2 = 0.06; r2 = 0.13), and large (η2, ηp2 = 0.14; r2 = 0.26). Magnitude of effect is demonstrated ↓ or ↑ = Small Effect, ↓↓ or ↑↑ = Moderate Effect, ↓↓↓ or ↑↑↑ = Large Effect

* = study presented no effect size for the finding

As exercise intensity increased, the concentric phase of all lifts were prolonged [23, 30, 31, 34, 36], with concurrent increases in peak and mean force production [41, 42], and decreases in mean and peak velocity [4, 17, 20, 26, 30, 31, 33–35, 40, 41]. Carroll et al. [26], however, found mean velocity varied considerably between individuals at heavier loads (coefficient of variation (CV) = 10–55%).

The relationship between power and intensity varied by lift. The back squat showed decreases in power with increasing intensity [26] with substantial between-subject variability (CV = 19–64%). In contrast, deadlift studies showed that peak power occurred at different intensities depending on measurement focus: Blatnik et al. [41] found system (bar + body) peak power was maximized at 70% 1RM, though bar and body peak power independently decreased. Lawson et al. [42] identified 90% 1RM as optimal for peak force and RFD. These findings highlight that power output varies by lift, with back squat power decreasing with increasing load and demonstrating substantial between-subject variability, while deadlift power appears to be maximized at around 70–90% of 1RM.

Phase-specific kinematic changes were observed. At heavier intensities, the SR and AR in both the back squat and bench press exhibited increased absolute and relative changes in duration [3, 23, 34, 36], while the DR and MSR phases showed relative decreases [23, 36]. Notably, in the squat, Larsen et al. [34] reported unchanged SR initiation but delayed transitions in the second half of the SR, reflecting increased time spent accelerating the barbell with intensity [36].

Joint coordination and segmental variability increased with intensity, with Maddox et al. [4] and Bryanton et al. [17] observing a greater focus on knee extension earlier in the AR of the squat, rather than the hips and ankles. In the squat, this was evidenced by decoupling and increased coupling angle variability between the knee–hip and thigh–trunk segments, occurring specifically during the SR and AR [4]. Consequently, as relative load increases during the squat, the knees extend disproportionately more than the hips, resulting in greater trunk forward lean during the early concentric phase [17, 20], and increased horizontal barbell displacement [17, 23]. Similarly, higher loads were also associated with more horizontal displacement in the bench press [3].

Force requirements changed at specific joints with load. Bryanton et al. [17] and Maddox et al. [4] found that in the back squat, while knee extensor net joint moments (NJMs) remained relatively stable across intensities, ankle plantar flexor and hip extensor NJMs increased. Bryanton et al. [17] further observed that knee extensor NJMs were strongly influenced by squat depth, with greater values at deeper positions, and that hip extensor NJMs also increased with depth. These findings suggest that under heavier intensities, joint contributions shift toward the hips and ankles, while the knee’s role remains relatively constant across loads but increases with depth. Overall, the concentric phase, especially the AR and SR, exhibited the most prominent load- and depth-dependent kinematic and kinetic changes.

### Relationship between Fatigue and Kinematics and Kinetics

Fatigue was examined in the back squat [14, 16, 33, 35] and bench press [18, 37, 39]. Across the included studies, fatigue was inferred through one of three primary methods: comparisons between different repetition maximum ranges [18, 33], varying proximities to failure [14, 35, 37], or comparisons between the initial and last repetition within a multi-repetition set [16, 39].

Fatigue-induced changes included decreases in mean and peak velocity [18, 37] and increases in repetition duration [39]. Mean and peak power declined across repetitions in the squat [16, 35], and total work decreased in the bench press [37]. Furthermore, a study by Weakley et al. [14] found that greater velocity loss thresholds were associated with reductions in peak power, while mean force production remained statistically unchanged.

Exercise-specific adaptations were evident. In the back squat, Brice et al. [16] found decreases in joint-specific angular velocity and power at the hips, knees, and ankles under fatigue. Van Den Tillaar and Saeterbakken [39] similarly reported increased barbell displacement, associated with changes in altered neuromuscular activation. While another study by Larsen et al. [18] found that positional changes weren’t effected during the first and final repetition within a set. These studies highlight the differential effects of fatigue across exercises, with the back squat showing more pronounced biomechanical alterations compared to the bench press.

Overall, both increased intensity and fatigue led to declines in barbell velocity and increases in repetition duration, while fatigue caused a decrease in peak and mean power output. Furthermore, fatigue may induce more pronounced positional and mechanical changes in the barbell squat compared to the bench press.

## Discussion

The review aimed to synthesize current research on the effects of intensity and fatigue on the kinematic and kinetic characteristics of performance of the barbell back squat, bench press and deadlift in experienced lifters. To date, no systematic review has investigated how varying levels of intensity or fatigue result in consistent and observable differences in trained lifters, specifically in back squat, bench press, and deadlift.

The primary finding of this review was that increases in intensity and fatigue consistently led to decreased mean and peak barbell velocity in the back squat and bench press [26, 30, 38, 39], along with increases in lifting time (particularly in the concentric phase) [4, 23, 36, 39]. A key distinction between intensity and fatigue lies in their effects on force-time characteristics. Increased intensity elevated force production [41, 42], while having a less clear relationship with power [26, 41, 42]. Alternatively, fatigue led to decreases in mean and peak power in the squat [14, 35] and work in the bench press [37], while a study by Weakley et al. [14] demonstrated non-significant changes in mean force production in the squat between 10%, 20%, and 30% velocity loss thresholds. These findings suggest that, unlike intensity, fatigue does not consistently reduce force production. Therefore, the acute effects of fatigue on force output warrant further investigation to clarify the conditions under which force production is preserved or impaired during the barbell squat, bench press, and deadlift [14, 35, 37].

In contrast to velocity and force, power production demonstrates a more inconsistent relationship with intensity. Blatnik et al. [41] found that peak barbell power occurred at 50% 1RM, Lawson et al. [42] found that RFD had no significant difference between loads, and Carroll et al. [26] found a negative linear relationship between power and intensity with a coefficient of variation ranging from 19.2% to 64%. This suggests that intensity may impact how much total force is being produced, but the rate at which force is produced has yet to be fully understood. Given these inconsistent findings regarding power production and RFD, further research on experienced lifters is needed to clarify the relationship between intensity and power, while more conclusive evidence supports force production, velocity, and lifting duration as key performance indicators.

Kinematic (e.g., velocity, movement duration, joint position) and kinetic (e.g., joint loading) characteristics varied across concentric subphases (AR, SR, MSR, and DR) [4, 23, 36, 39]. Higher intensities increased the duration to reach peak velocity [31], consequently increasing time spent in the sticking and acceleration regions [23, 36]. These changes are often accompanied by altered movement strategies, such as increased forward torso lean in the squat [4], or bar path that drifts toward the shoulders in the bench press [3, 23], which coincide with shifts in horizontal barbell displacement for both the squat and bench press [3, 17, 20, 33, 36, 40]. As intensity and fatigue increase, greater stress is placed on larger joints, particularly the hips (in back squat) and shoulders (in bench press) [4, 16, 17, 30, 33, 36]. These compensatory shifts may partially explain the increased force production and reduced power observed at higher intensities, highlighting the need for practitioners to monitor these changes and implement targeted assistance exercises to address altered joint contributions.

Findings related to the eccentric, lowering, phase of the lifts were mixed between back squat and bench press. Van Den Tillaar et al. [31] reported that eccentric phase duration remained unchanged across intensities in the squat, whereas Van Den Tillaar and Sousa [38] observed increases in duration in the bench press. Although not included in this review, findings from Carzoli et al. [43] further support these differences by reporting that normative eccentric duration increased with load in both lifts, but tended to be lower in the bench press (100% vs. 60% of 1RM) compared to the squat, which was performed faster at higher intensities (60%, 80%, and 100% of 1RM), particularly under heavier loads. These findings suggest that lifters may adopt more conservative eccentric strategies during the bench press at heavier loads.

Inter-individual variability in kinematic (e.g., velocity and joint mechanics) and kinetic (e.g., power) responses to increased intensity was only addressed in a few studies within this review [4, 26, 42]. While load-induced changes tended to follow predictable trends, individual differences became more variable at higher intensities [4, 26]. This pattern is consistent with broader observations in resistance training literature, where lifters adopt varied movement strategies and joint loading patterns under high mechanical demand, even when performing the same exercise at similar intensities [24, 44]. Elliott et al. [23] and Maddox et al. [4] reported that lifters manage increased joint torque at higher intensities by altering bar path and relying more on larger proximal joints. In contrast, Larsen et al. [18] and Brice et al. [16] found that technique remained stable, but joint moments shifted, specifically, with increased hip force and decreased knee force during the back squat. Collectively, these findings highlight substantial inter-individual variability in responses to load. However, the extent to which factors such as training status, structural characteristics, or sex contribute to this variability remains unclear—important questions for future research.

### Methodological Considerations

This systematic review followed PRISMA guidelines and was pre-registered in PROSPERO. Eligible studies were cross-sectional, assessed free-weight back squat, bench press, or deadlift, and included trained participants aged 18 or older. We restricted inclusion to studies that directly compared the effects of intensity or fatigue on kinetic or kinematic outcomes in these lifts. This was done to improve the generalizability of findings, as such studies are more likely to be adequately powered, explicitly designed to isolate the effects of fatigue or intensity, and report statistical comparisons across relevant conditions. As a result, some studies with alternative designs or broader aims despite containing relevant data may have been excluded.

Two authors independently conducted screening and quality assessment using the NIH Quality Assessment Tool for Observational Cohort and Cross-Sectional Studies. Seventeen of the 22 studies were rated as “good” quality (≥ 6 criteria met), while five [3, 4, 20, 23, 36] were rated “fair” or “poor” due to insufficient reporting of sample size or recruitment details. While all included studies defined their protocols and outcomes clearly, several limitations should be noted: (1) restrictive inclusion criteria may have excluded potentially relevant studies; (2) only two studies [14, 26] controlled for confounding factors; and (3) only five studies conducted a priori sample size calculations [16, 17, 33, 34, 37], limiting generalizability for the results of these studies.

In addition, many included studies did not clearly describe how participants were recruited or what inclusion criteria were applied, with only seven studies providing detailed recruitment strategies [14, 30, 31, 34, 35, 38, 42]. This is a critical limitation, especially given the review’s focus on consistent mechanical responses in trained individuals, where variability in training background, experience level, or competitive status could significantly influence kinetic and kinematic outcomes. Without transparent reporting, inter-study variability in training background or competitive experience may obscure true mechanical patterns. Moreover, the included studies did not account for intra-subject variability, such as individual shifts in joint coordination or force output across increasing intensities, which may further contribute to inconsistent findings. Future studies should prioritize detailed participant selection reporting to strengthen comparability and interpretation.

## Conclusions

This review indicates that intensity and fatigue significantly influence kinetic and kinematic outcomes in the back squat and bench press, with consistent decreases in barbell velocity and increased lifting time at higher intensities. While intensity reliably increased force output, the effects of fatigue on force were more variable. Findings also revealed notable phase-specific changes, especially during the AR and SR. However, results for power output were inconsistent, and limited data were available for the deadlift and for studies using rigorous recruitment or confounder control. More research is needed to explore both inter- and intra-individual variability in movement kinetics and kinematics, to better inform individualized coaching and clinical decisions when instructing on powerlifting exercises.

## Supplementary Information

Below is the link to the electronic supplementary material.

Supplementary Material 1

Supplementary Material 2

Supplementary Material 3

Supplementary Material 4

## Data Availability

Data used for this systematic review will be made available on inquiry.

## Abbreviations

EMG: Electromyography
1RM: 1-repetition max
RPE: Rating of perceived exertion
RIR: Repetitions in reserve
AR: Acceleration region
SR: Sticking/failure region
MSR: Maximum strength region
DR: Deceleration region
PRISMA: Preferred Reporting Items for Systematic Reviews and Meta-Analyses
PROSPERO: International Prospective Register of Systematic Reviews
MANOVA: Multiple analysis of variance
CV: Coefficient of variation
